# Student evaluation of the impact of changes in teaching style on their learning: a mixed method longitudinal study

**DOI:** 10.1186/s12912-018-0293-z

**Published:** 2018-06-15

**Authors:** Susan Jones, Somasundari Gopalakrishnan, Charles A. Ameh, Brian Faragher, Betty Sam, Roderick R. Labicane, Hossinatu Kanu, Fatmata Dabo, Makally Mansary, Rugiatu Kanu, Nynke van den Broek

**Affiliations:** 1 School of Human and Health Sciences, University of Huddersfield, Queensgate, Huddersfield, HD1 3DH UK; 2 Centre for Maternal and Newborn Health, Liverpool School of Tropical Medicine, Pembroke Place, Liverpool, L3 5QA UK; 3 Centre for Maternal and Newborn Health, Liverpool School of Tropical Medicine, Wilkinson Road, Freetown, Sierra Leone; 4 Welbodi Partnership, Ola During Children’s Hospital, Freetown, Sierra Leone; 5 Ministry of Health and Sanitation, Youi Building, Freetown, Sierra Leone; 6 School of Midwifery, Makeni, Sierra Leone

**Keywords:** Nurse education, Student satisfaction

## Abstract

**Background:**

Maternal and Child Health Aides are the largest nursing cadre in Sierra Leone providing maternal and child health care at primary level. Poor healthcare infrastructure and persistent shortage of suitably qualified health care workers have contributed to high maternal and newborn morbidity and mortality. In 2012, 50% of the MCHAides cohort failed their final examination and the Government of Sierra Leone expressed concerns about the quality of teaching within the programmes. Lack of teaching resources and poor standards of teaching led to high failure rates in final examinations reducing the number of newly qualified nurses available for deployment.

**Methods:**

A mixed-methods approach using semi-structured observations of teaching sessions and completion of a questionnaire by students was used. Fourteen MCHAide Training Schools across all districts of Sierra Leone, 140 MCHAide tutors and 513 students were included in the study. In each school, teaching was observed by two researchers at baseline, 3 and 6 months after the tutor training programme. Students completed a questionnaire on the quality of teaching and learning in their school at the same time points.

**Results:**

A total of 513 students completed the questionnaire, 120 tutors took part in the training and 66 lessons across all schools were observed. There was a statistically significant (*p* < 0.05) improvement in mean student evaluation of teaching and learning in 12/19 areas tested at follow-up compared to baseline. Observation of 66 teaching sessions demonstrated an increase in the number of student-focused, interactive teaching methods used.

**Conclusion:**

Prior to the teaching and learning workshops there was little student-focused learning within the schools. Teaching was conducted predominantly using lectures even for practical sessions. Training tutors to move away from didactic teaching towards a more student-focused approach leads to increased student satisfaction with teaching and learning within the schools.

## Background

Sierra Leone is ranked 183rd among 187 nations in the Human Development Index and the country faces many challenges in providing maternal and newborn care. For a population of just over 6 million, there are 0.22 nurses-midwives and 1.66 medical doctors per 10,000 population. This demonstrates a lack of human resources when compared to the World Health Organization recommendation of 23 doctors, nurses and midwives per 10,000 population [1]. In 2000 Sierra Leone reported a maternal mortality ratio (MMR) of 1800 per 100,000 live births which improved steadily to 1495 in 2005 and 857 in 2008. However, in 2016, post the Ebola epidemic, there was again a rise in MMR to 1360, compared to the sub-Saharan African average of 546/100,000 [1].

Following the introduction in Sierra Leone of free health care for pregnant and lactating women in 2010, there has been a steady increase in the number of women attending for antenatal care, with 78% of women attending for 4 visits and nearly 97% attending at least one antenatal care (ANC) visit [1]. In working towards Millennium Development Goal 4 (to reduce child mortality by 2015) and 5 (to improve maternal health by 2015) Sierra Leone instituted measures to improve the number of deliveries attended by a skilled birth attendant (SBA). In 2000, 42% of births were attended by an SBA which rose to 97% in 2012 but dropped back to 60% in 2016 [2]. The causes of maternal death are well known with 80% being due to haemorrhage, infection, eclampsia, complications of unsafe abortion and obstructed labour [3]. |It is also well known that having healthcare workers who are skilled in providing the continuum of maternal and newborn care and are working in an enabling environment significantly reduces both newborn and maternal morbidity and mortality.

The shortage of healthcare workers in Sierra Leone has led to the introduction of new cadres of nurses. Maternal and Child Health Aide (MCHA) training was introduced in 1972 in four districts. Women who had at least 3 years of secondary education and were aged over 25 years of age were able to apply for training [4, 5]. A gradual roll-out of the programme meant that in 2012 all 14 districts of Sierra Leone had a MCHA training school. The most recent intake in 2012 saw a total enrolment of 750 students across all districts. In 2014 MCHAs constituted 46% of the total workforce for health, and provided the majority of maternal and newborn health care. Strengthening pre-service training to maximize the learning opportunities of MCHA is crucial if good quality maternal care is to be provided [6].

This study aimed to determine the impact of a tutor development programme on student satisfaction with teaching and learning. In 2012, a high failure rate (50%) of the MCHA student cohort led the Ministry of Health and Sanitation (MoHS) and partners to question how effective the teaching and learning was within the schools. With its funding partner, UNICEF, the MoHS requested the evaluation of the academic quality of the MCHA training programme including the involvement of the programme managers and staff responsible for the teaching and learning environment.

An observational study of the teaching styles employed within each MCHAide school in 2015 showed teaching was predominantly didactic, teaching aids were rarely used (less than 15%) and teaching was tutor focused rather than student focused [7]. Over-crowding in many of the schools, lack of appropriate classroom furniture and inconsistent electricity supply where findings contributing to a poor learning environment and academic output in these schools.

Studies have shown that there is a positive correlation between the quality of teaching and student satisfaction [8] and student satisfaction and grades [9]. Good teaching quality enhances student performance leading to higher scores and higher student satisfaction [10]. We therefore, looked at student satisfaction as an indicator for changes in the quality of teaching. This allowed us to determine quality of teaching without adding the additional burden of further assessments to the students and the school. Results from the student final examinations were also recorded as a measure of student performance.

The research team were asked to work with the MCHA schools to improve the quality of teaching and the learning environment in the Schools. The aims of this study were: 1) to examine the impact of a training programme to equip tutors with the skills to increase the number of student-focused lessons; and 2) to determine the impact of changes in teaching on student satisfaction.

Style of teaching, subject matter and student learning styles all combine to influence how well a student learns and how satisfied they will be with their education [11]. The high failure rate in the 2012 MCHA cohort of students indicates a breakdown in one or more of these areas. For nursing students to become competent and independent practitioners who can recall theory and practice in sometimes stressful situations, requires an education system that encourages student focused learning, reflective practice and takes account of the diversity of the teaching matter [12]. The global shift from traditional lectures to interactive and group based learning is based on the social constructivist theory that learners build on the experiences, previous knowledge, cultural backgrounds and social influences to become self-directed learners. In Sierra Leone students are used to being taught through a reductionist teacher focused approach; it is therefore important to understand how any changes in teaching practice influence student satisfaction and learning.

Concerns about the quality of pre-service education are not confined to Sierra Leone. Fullerton et al. [13] report on three sub Saharan African countries which continue to use didactic, lecture based training despite ongoing concerns about the quality of such training. As in the MCHA schools many of the teachers that Fullerton observed had no formal training in teaching and learning and were employed for their clinical rather than teaching expertise.

## Methods

This longitudinal, mixed methods study used a phenomenographical approach which offers a more sophisticated and complex understanding of teaching and learning because it includes what is happening with both the tutors and the students [14–16]. Structured non-participant observation was used to observe the amount of student focused teaching in each school at baseline.

### Participants

All students from all 14 MCHA schools were asked to participate in the study. Given the large number of students (750) self-administered questionnaires were used to determine student satisfaction with the learning and teaching environment. Each school takes approximately 40–60 students in each cohort with a total of 1258 h of taught theory from 10 teachers per school. At the request of the MoHS, all 14 schools were included in the programme to prevent any school being potentially disadvantaged. In addition to the tutor training, each school received skills room equipment and audio-visual aids to assist tutors in developing more interactive teaching methods.

### Data collection

From 2013 to 2014 four education experts from the research team facilitated four district based, 2-day workshops for 10 core teaching staff (including the School coordinator) from each of the 14 MCHA schools (140 core staff in total). There were 35 tutors in each work shop. None of the MCHA tutors had a formal teaching qualification.

The workshop aimed to develop tutors understanding of teaching and learning theories and how to apply these in practice; to understand the concept of student focused learning; to understand how to apply different teaching methods within the classroom and to develop skills in reflective practice. The workshops covered active teaching and learning methods, lesson planning, writing learning outcomes, reflective practice, supportive supervision, mentorship and effective learning environments. During the final session of the 2-day workshop tutors developed an action plan for their own school to implement their learning from the workshop into practice. In addition, tutors also took part in formative teaching practice both in the classroom and simulation or skills room.

All workshops were completed within a two-week period and first follow up observations conducted 3 months afterwards. It was anticipated that this would allow tutors time to start implementing changes in some of their lessons. Observed classes included only those tutors who had been to the workshop.

A descriptive analysis of the age and entry qualification of MCHA students at the time of the study was conducted by reviewing admission records. This was to establish compliance with admission criteria as there is a high potential that less qualified students (on entry) may find it more difficult to cope with the curricula.

Prior to the tutors training programme a baseline visit was conducted for each of the 14 MCHA schools in August–September 2013. Three follow-up visits were planned to all 14 MCHA schools across Sierra Leone. The follow-up visits were timed to coincide with key dates during the MCHA training.

First follow-up was at the end of the students first year (3 months from baseline). Second follow-up occurred immediately prior to their second year examinations (6 months from baseline). Final follow-up was conducted at the end of the programme (12 months from baseline).

Due to the Ebola epidemic in Sierra Leone which occurred from May 2014 to December 2015 the MCHA schools were closed in August 2014. Only 10 of the 14 schools could be visited for the second follow-up and no schools could be visited for the third follow-up. Final results are based on the 10 schools seen at second follow-up only.

At each visit (baseline, 3 months and 6 months), both qualitative and quantitative methodology was used to gain as full a picture as possible of the teaching and learning within each school and allow for triangulation of data. Data collectors worked in pairs to administer the student questionnaire but conducted independent observation of the teaching using a standardised data collection tool.

The aim was to observe two one-hour teaching sessions at each visit in each MCHA school (2x14x3 visits = 84 observations) and ask 750 students to complete a questionnaire at each visit (3 × 750 = 2250 questionnaires). Outcome measures were student evaluation of teaching and learning an increase in student focused sessions, reduction in the use of didactic teaching methods and increase in student learning.

Both tutors and students were provided with written and verbal information about the study by the research team and asked to sign a consent form if they agreed to participate.

#### Observation of teaching

A structured non-participant observation method was used to observe teaching sessions in each school at each visit [17]. A full explanation was given to the MCHA tutors on the purpose of the planned visits to their schools by the research team and their consent for this sought. Event sampling was used to select the teaching sessions and keep disruption of the normal school timetable to a minimum [18]. There is an underlying assumption when using structured observation that the researchers are familiar with, and understand, the activity being observed [19]. Each member of the research team was involved with and experienced in teaching and learning at a pre and/or post registration level. A modified pre-designed structured observation form was used that assessed teaching methods, student learning and student involvement.

Researchers also took notes during the observations which were subsequently transcribed. Transcription was completed by the same members of the research team to aid with familiarisation of the data which is a key aspect of the framework approach [20]. One member of the research team independently coded the transcripts from four schools which gave an initial coding framework. Though a deductive approach was used to provide an initial structure for the observations an inductive open- coding approach was taken for the final coding [20]. Informal feedback was provided to tutors after the observations by the research team.

#### Student questionnaires

Students were asked to evaluate the teaching and learning within their own school through an anonymised self-administered questionnaire. Questionnaires were adapted for language and clarity in partnership with MoHS from those used at the Liverpool School of Tropical Medicine to obtain student feedback on teaching. Students rated the lessons in three areas, teaching methods, student learning and student involvement, as these are thought to be the key components which influence teaching and learning [Clark 11]. A 19 question, 5-point Likert scale was used. Basic demographics including the student’s highest academic qualification on entry to the programme and their age were also obtained. Students completed the questionnaire immediately after the lesson being observed. MCHA tutors were asked to leave the classroom during the completion of the questionnaire.

### Data analysis

Qualitative data analysis was completed using the Framework Analysis approach. This approach is useful where data covers similar topics or key issues and so can be categorised [20]. In this study the structured observation form used a deductive approach to pre-select key themes of teaching and learning which could be considered as the key issues [20]. It was expected that other sub themes may also occur at follow-up visits which would be incorporated into the Framework analysis.

All student questionnaires were electronically scanned and processed using Formic^®^. SPSS version 22 was used for analysis. New random samples were selected on each occasion, so most students completed a questionnaire at just one of the three assessment times; these samples were considered to be statistically independent. One-way analyses of variance (ANOVA) were used to evaluate the changes in mean student satisfaction scores across the three assessment points; statistical significance was set at the conventional alpha level of 5% (*p* ≤ 0.05). Each of the test items (questions) were analysed individually and then the total score across all 19 items was evaluated; Cronbach’s alpha (coefficient of reliability) was calculated to ensure adequate internal reliability of this total score.

## Results

### Student evaluation of teaching and learning

Five hundred thirteen students completed the questionnaire at baseline; 518 at first follow-up and 466 at second follow-up. All students on the MCHA programme are female. Descriptive analysis of the age and qualification on entry showed that not all students met the admission criteria of; 1) being between the ages of 26–40 years; 2) have completed level-3 at senior secondary school, and; 3) at least attempted but not necessarily passed the West Africa Senior School Certificate Examination (WASSCE) or GCE O-level (Table 1). Sixty-three (12.5%) students had failed to attain the minimum academic entry criteria. In total, 44% (222) had a higher qualification than WASSCE on entry to the programme. Of the 504 students who completed the baseline questionnaire, 276 (55%) had not passed their final examination at the end of their secondary school education.

**Table 1:** Age and qualification of students on entry to Maternal and Child Health Aide training

Age at entry n (%)					
Student age in years	18–25	26–33	34–41	42–50	Meet criteria n (%)
	108 (21%)	230 (45%)	146 (28%)	20 (4%)	376 (75%)
Educational qualification at entry n (%)					
SSS3	Passed WASSC	Attempted WASSC	GCE O level	Other	Meet criteria n (%)
63 (12.5%)	6 (1%)	213 (42%)	133 (26%)	89 (18%)	441 (88%)

*SSS3* 3rd year of senior secondary school, *WASSCE* West Africa Senior School Certificate Examination

Positive evaluation of teaching and learning by the students increased from baseline to second follow up as the number of student focused lessons increased. Twelve (1) out of 19 areas assessed obtained a statistically significant higher mean score (*p* < 0.05). Only 4 areas did not show an increase in mean scores over time (Table 2). In particular students positively evaluated the usefulness of teaching methods (*p* = 0,002) as more student focused methods were used.

**Table 2:** Student assessment following programme to strengthen teaching at MCHAide schools across Sierra Leone

Name of variable in student questionnaire	Baseline			3-month follow-up			6-month follow-up			*p* value*
	Number of students *n*	Mean score (1–5)	95% CI	Number of students *n*	Mean score (1–5)	95% CI	Number of students *n*	Mean score (1–5)	95% CI	
1. Clarity of objectives	509	4.72	4.66–4.79	516	4.79	4.73–4.85	466	4.80	4.75–4.86	0.107
2. Easy explanations given	510	4.67	4.61–4.73	516	4.78	4.73–4.83	465	4.80	4.75–4.85	0.003
3. Useful teaching methods used	510	4.52	4.44–4.60	516	4.71	4.65–4.77	466	4.75	4.70–4.80	0.002
4. Tutor encourages questions	510	4.73	4.67–4.79	517	4.83	4.78–4.87	466	4.81	4.76–4.85	0.066
5. Lesson is well organised	506	4.33	4.23–4.43	518	4.72	4.67–4.78	464	4.68	4.62–4.73	< 0.001
6. Tutor encourages thinking	508	4.28	4.19–4.37	515	4.55	4.48–4.62	465	4.55	4.49–4.61	< 0.001
7. Tutor is available for extra support	512	3.65	3.52–3.78	515	3.93	3.82–4.05	464	4.00	3.88–4.11	0.001
8. Tutor treats students respectfully	512	4.38	4.27–4.48	515	4.28	4.18–4.38	465	4.56	4.49–4.63	0.005
9. Wide breadth of learning	513	4.64	4.58–4.71	518	4.81	4.76–4.85	464	4.83	4.79–4.87	< 0.001
10. Level of student Interest in subject	511	4.63	4.56–4.69	518	4.73	4.68–4.79	463	4.75	4.69–4.80	0.011
11. Students think subject taught is important	513	4.89	4.85–4.93	518	4.90	4.87–4.93	463	4.88	4.85–4.92	0.402
12. Students attend all lessons	505	4.64	4.57–4.71	516	4.61	4.54–4.67	462	4.59	4.53–4.65	0.004
13. Students complete homework	496	3.85	3.73–3.97	510	4.24	4.16–4.33	459	4.33	4.26–4.40	< 0.001
14. Students participate in class discussions	509	4.67	4.62–4.73	512	4.68	4.63–4.73	463	4.68	4.63–4.74	0.706
15. Students expectations of breadth of learning	510	4.56	4.48–4.64	513	4.81	4.76–4.85	463	4.81	4.77–4.86	< 0.001
16. Students think lessons are difficult	501	2.98	2.85–3.11	509	2.62	2.49–2.74	457	2.86	2.73–3.00	< 0.001
17. Students find time for Self-study	500	3.88	3.76–4.00	513	3.85	3.74–3.96	463	3.95	3.85–4.06	0.673
18. Students find it difficult to find time for self-study	507	3.02	2.88–3.16	514	2.74	2.61–2.87	463	3.11	2.98–3.24	< 0.001
19. Hours of self-study by student	511	2.93	2.84–3.02	517	2.81	2.73–2.89	464	2.87	2.79–2.96	0.239
Total (out of 95)	460	71.3	70.61–72.03	471	73.6	73.10–74.12	445	73.8	73.24–74.36	< 0.001

*p value for comparison of scores at follow up with those obtained at baseline

Variables which could be considered to be important in developing more student-focused learning and therefore to be influenced by the training workshop included; the teaching methods used, being encouraged to think and to ask questions, the availability of tutors outside of lessons and the breath of learning and student interest in the subject. There was a statistically significant increase in student scores at follow-up compared to baseline. Students also reported that lessons were better organised and tutors more respectful to their students. A decrease in the number of students reporting that lessons were difficult from baseline to follow-up may indicate the positive impact of the new teaching styles on student learning as they moved into their second year.

There was a statistically significant increase in the positive evaluation by students of variables which could be considered to be indirectly influenced by what goes on in the classroom; these included students completing homework and the numbers attending all lessons. Students continued to report that it was difficult to find time for self-study which may be influenced by commitments outside of the school including family.

Cronbach’s alpha for the total score using all 19 questions was 0.71; this increased to 0.72 when the question on ‘Hours of self-study’ was not included and to 0.79 when the questions on ‘Content of lesson too hard’, ‘Hours of self-study’ and ‘Difficult to find time’ were not included. This total score increased highly significantly between baseline and the first follow-up assessment but there was only a very modest and non-significant increase between the first and second follow-ups.

### Observation of teaching

Across all 14 schools 120 85%) tutors took part in the teaching and learning workshop. A total of 26 lessons were observed at baseline, 21 at the first follow-up and 19 at the second follow-up (66 of 84 planned observations = 78.6%). Following transcription, qualitative data was coded using Framework analysis into a total of 10 key themes and 48 sub themes (Table 3).

**Table 3:** Observation of teaching: key themes and sub themes identified using framework analysis

Key Theme	Sub themes
Student participation	• use of local language • role play • examples from students • answering questions • asking questions • student reflection • students summarise key points of lecture • note taking • student tasks
Lesson preparedness by tutor	• lesson notes/guide • learning outcomes • teaching plan • adherence to timetable • organization • pace of teaching • Interruption of session by tutor
Use of visual aids/teaching equipment	• blackboard • IT • diagrams • use of equipment/anatomical models
Depth of learning	• memorisation • rote learning • understanding • synthesising information • meeting learning outcomes/objectives
Style of teaching	• lectures • discussions • links to future lectures • links to previous lectures • practical demonstrations • links to practice • scenarios
Provides students with feedback	• individualized feedback given • lack of formative feedback • student peer feedback given
Content	• depth • appropriateness • repeated topic • did not complete content
Assessment	• formative • summative
Teaching environment	• ventilation • light • space • desks and chairs available • adapted teaching style to fit environment
Student/tutor relationship	• student commitment • tutor commitment • student/tutor rapport • control of class

#### Student participation, teacher preparation and style of teaching

Observed methods of teaching were classified into two groups based on the amount of student involvement (Table 4). Tutor-focused lessons had minimal student involvement, relied on didactic methods and centred on students answering questions as a group rather than as individuals. Student-focused lessons were those which encouraged individual questions and answers and employed interactive teaching methods such as role play, discussion groups or practical’s. At baseline 58% out of the 26 lessons were described as using didactic (tutor-focused) methods, this decreased to 33% of lessons at first follow-up and to 31% of lessons at second follow-up. There was a corresponding increase in student-focused (interactive) lessons from 4% at baseline, to 29% at first follow-up and 21% at second follow-up. The number of lessons described as being well organised also increased from baseline to follow up.

**Table 4:** Classification and characteristics of observed teaching styles

Didactic/Tutor-focused	Interactive/Student group-focused
Lecture	Student practical
Group Q&A	Scenarios
Tutor demonstration	Role play
	Seminar discussion
	1:1 tutoring
	Individual Q&A
	Tutorial

#### The learning and teaching environment

The quality of the learning and teaching environment varied across the schools with some having well ventilated, well-lit classrooms with electricity and of an adequate size, to those that were too small for the number of students, poorly ventilated and without electricity. However, following the training workshops tutors did try and make better use of the limited space and facilities they had and included more student-focused teaching such as group work and discussions. Despite the many challenges that the schools face there were good relationships in each school between the students and their tutors, particularly with the co-ordinators for each school who also provided the majority of teaching.

Each training schools was provided with audio visual equipment (projectors, a desktop and laptop computer) and skills equipment including mannequins and anatomical models to develop a skills room.

#### Lesson content, depth of learning and student feedback

The depth of student learning was found to be an important issue, especially at baseline where the majority of teaching was didactic with rote learning, memorising and little synthesising of information. Tutors sometimes ran out of time to meet all of the lessons learning outcomes or were repeating previous lessons when timetabled tutors did not attend their sessions. A lack of individualized student feedback was noted, with peer feedback given only in the form of applause by the whole class in any particular session. No formative or summative assessments were observed during the study.

## Discussion

Teaching and learning is a complex mix of many components which all contribute to the overall learning experience of the student. Studies suggest that students learn in different ways and that tutors should adopt a teaching methodology that takes into account these different learning styles [11]. Matching teaching styles with each student’s individual learning style can be difficult if not impossible for large student groups. Using diverse teaching styles is therefore important in order to maximize learning [12]. In many countries teaching has moved away from didacticism to a more facilitative approach which sees the student rather than the tutor as the focus of the classroom. The aim of such a student-focusedapproach is to equip students with skills in critical thinking, problem-solving and independent learning [21]. This is particularly important in dynamic areas of work such as nursing. Discerning how students evaluate the impact of changes in teaching styles is important to fully understand their impact on learning. Changes in teaching style are not just challenging to faculty but also to students and this has to be acknowledged if they are to be effective. In this study students recognized the changes in teaching style and positively evaluated their usefulness to their learning.

Some argue that the most effective teaching style is the one that the tutor feels most comfortable using, despite the students learning style. However, teaching activities and styles that can generally be classified as being ‘active teaching methods’ have a more positive impact on students learning outcomes [22]. In nursing using a didactic, information-giving teaching approach does not equip students with the ability to question and synthesize multiple sources of information, skills which will be needed by nurses in their daily practice [23].

The introduction of task shifting and of new cadres of healthcare workers in Sierra Leone has meant that new training schools have been set up; bringing challenges in finding suitably qualified tutors to deliver a new and expanded curriculum in often shorter than conventional time frames. Task shifting generally involves shorter, more focused training programmes to equip healthcare workers for specific settings [24]. Sierra Leone is not unique within Africa in providing education from junior to higher education through a didactic teaching process [25]. There is a need to develop an education system that provides training for the tutors (often senior healthcare providers) whose capacity and enthusiasm to deliver the new curriculum using new teaching methodology is critical to the success of the programmes. In addition, in most settings, there is an identified urgent need to improve the quality of the learning environment [26, 27].

### Main findings

Both tutors and students contribute to the success of teaching and learning. From a tutor’s perspective how they engage and work with students can contribute to the overall effectiveness of their teaching. The workshop intervention was deemed successful in encouraging tutors to change their methods of teaching with a decrease in tutor-focused and an increase in student-focused methods of teaching. The aim of the training workshop was to train and encourage tutors to use a wider variety of teaching methods and move away from simple didactic teaching, and in this respect the workshop was successful. However, this change in teaching also needs to be recognised as being useful by the students for their learning.

Data from the student questionnaires showed a positive evaluation of teaching and learning in the schools as the number of student focused lessons increased. Interactive teaching methods have increased in popularity in high resource areas but less so in low resource areas such as Sierra Leone. Where there is a lack of education and information technology resources then the teaching methods used take on an even higher importance as they are often the only information resource available to students. Encouraging students to be part of developments in teaching by asking them to evaluate new methods can further help to develop the learning and teaching environment.

Though there is an agreed minimum qualification for entry to the MCHA training programme in Sierra Leone, 12.5% of the students did not have this and this may have affected their ability to learn at the required level. At follow-up there was a decrease in students who reported finding the lessons difficult (mean 2.98 at baseline compared to 2.86 at second follow up follow-up). Despite the difficulty of lessons increasing as the students moved into their second and final year when more complex subjects were taught. This may be a reflection of the impact that the move from didactic to student focused teaching was having with students reporting an increase in being encouraged to think, ask questions and a greater breadth of learning.

Some areas of learning and teaching are less easy for tutors to influence (hours of self-study, finding time to study) and it is reasonable to expect that just changing the methods of teaching could not influence these. Nevertheless, it is important for the overall picture of teaching and learning within the MCHA schools to know if there are factors outside of the classroom that may hinder learning. All of the students are female who have family commitments outside of their studies and which could reasonably be expected to impact on their self-study time. The majority of students lived in homes without a regular electricity supply further hampering study at home. Further research is needed to understand which particular enablers of student learning can be strengthened and how in this context.

Researchers were surprised to find that tutors advised students not to take notes. There is contradictory evidence on the effectiveness of in-class note taking and if this benefits students understanding or is a distraction. Though it would seem appropriate, given the lack of other reference material available, that the MCH Aide students should be allowed to take notes or have these provided by their tutors [28, 29]. Where students have limited or no access to learning materials or libraries the information provided within the classroom may take on a higher importance. The provision of learning resources is particularly important for this group of students who additionally reported finding time for self-study outside of the MCHA School difficult.

This study has important findings which need to be considered by countries looking at task shifting health care and introducing new cadres of nursing. The increase in numbers of healthcare workers in formal education programmes is not necessarily enough to meet the health needs of a country. The quality of the education programme and standards of teaching need to be addressed if such programmes are to successfully bridge the human resource shortages.

### Strengths and limitations

The study involved both tutors and students and therefore obtained a comprehensive view of teaching and learning within the schools. Observation of teaching was new to the MCHA tutors but appropriate within the context of the study to determine the styles of teaching used and was, in fact, welcomed by the tutors. The use of peer review of teaching (on which the observations were based) is well established in many academic institutions, the aim of which is to provide constructive feedback to tutors on their practice [30]. The presence of the observers may have altered the dynamics within the classroom [31].

A planned final follow-up visit at 12 months and focus groups with the tutors following tutor training could not be completed due to the Ebola epidemic which occurred from May 2014 to November 2015. This is unfortunate as it would have provided self-evaluation of the tutors and information on the sustainability of the observed changes within the schools. The researchers also planned to conduct student focus groups to explore their perceptions and experiences in more detail than can be done via a questionnaire, but again the Ebola epidemic prevented this.

## Conclusion

Encouraging student evaluation of new teaching styles is important if they are to engage fully with the learning environment. Tutors provide feedback to students on their progress but it is also essential for students to provide feedback on how teaching methods are impacting on their learning if nurse education is to progress and meet the global shortage of qualified nurses.

The aim of the tutor training workshop was to increase the standard of teaching and learning within the schools and increase the number of students passing their examinations. In the State final examination for the 2012–2015 cohort 80% of students passed compared to a 50% pass rate in the previous 2010 cohort (MoHS verbal communication from the national MCHA Coordinator).

## Abbreviations

ANC: Antenatal care
CMNH-LSTM: Centre for Maternal and Newborn Health at the Liverpool School of Tropical Medicine
MCHAides: Maternal and Child Health Aides
MMR: Maternal Mortality Ratio
MoHS: Ministry of Health and Sanitation
SBA: Skilled Birth Attendance
WASSC: West Africa Senior School Certificate Examination
